# Etiology of meningitis among adults in three quaternary hospitals in Mozambique, 2016–2017: The role of HIV

**DOI:** 10.1371/journal.pone.0267949

**Published:** 2022-05-11

**Authors:** Aquino Albino Nhantumbo, Charlotte Elizabeth Comé, Plácida Iliany Maholela, Alcides Moniz Munguambe, Paulino da Costa, Mariana Mott, Gabriella Rosa Cunha, Lúcia Chambal, Cícero Dias, Vlademir Vicente Cantarelli, Eduardo Samo Gudo

**Affiliations:** 1 Laboratório de Bacteriologia e Patógenos de Alto Risco, Instituto Nacional de Saúde, Ministério da Saúde, Maputo, Mozambique; 2 Instituto Nacional de Saúde, Ministério da Saúde, Maputo, Mozambique; 3 Unidade de Gestão de Dados, Instituto Nacional de Saúde, Ministério da Saúde, Maputo, Mozambique; 4 Universidade Federal de Ciências de Saúde de Porto Algre (UFCSPA), Porto Alegre, Brazil; 5 Departamento de Medicina at the Hospital Central de Maputo, Ministério da Saúde, Maputo, Mozambique; 6 Universidade Feevale, Novo Hamburgo, RS, Brazil; Vanderbilt University Medical Center, UNITED STATES

## Abstract

**Background:**

Meningitis remains an important cause of morbi-mortality in adults in sub-Saharan Africa. Data on the etiological investigation of meningitis in adults in Mozambique is limited and most studies were conducted in southern Mozambique. Identification of the etiology of meningitis in adults are crucial to guide prevention and treatments strategies. In this study, we determine the burden of fungal and bacterial meningitis among adults at the three largest hospitals in Mozambique.

**Method:**

We performed analysis of data from the routine sentinel surveillance system for meningitis in Mozambique from January 2016 to December 2017. Cerebrospinal fluid (CSF) samples were collected from eligible adults (≥18 years old) who met World Health Organization (WHO) case definition criteria for Meningitis. All samples were tested by cryptococcal antigen (CrAg) lateral flow assay (LFA), culture and triplex real-time polymerase chain reaction (qPCR) assay and all patients were tested for human immunodeficiency virus (HIV) using the national algorithm for HIV testing.

**Results:**

Retrospective analysis of 1501 CSF samples from adults clinically suspected of meningitis revealed that 10.5% (158/1501) were positive for bacterial and fungal meningitis. Of these 158 confirmed cases, the proportion of Cryptococcal meningitis and pneumococcal meningitis was38.6% (95% CI: 31.0% to 46.7%) and 36.7% (95% CI: 29.2% to 44.7%), respectively. The other bacterial agents of meningitis identified include *Neisseria meningitidis* (8.9%; 14/158), *Escherichia coli* (6.3%; 10/158), *Haemophilus influenzae* (5.1%; 8/158) and *S*. *aureus* (4.4%; 7/158), which represent (24.7%; 39/158) of the total confirmed cases.

**Conclusion:**

Altogether, our findings show a high burden of Cryptococcal meningitis among adults in Mozambique, especially in people living with HIV, followed by pneumococcal meningitis. Our findings suggest that rollout of CrAg Lateral Flow Assay in the health system in Mozambique for early detection of cryptococcus neoformans is necessary to improve overall patient care.

## Background

Meningitis remains among the leading causes of high morbidity and mortality in adults in Sub-Saharan African countries [1–5]. Even with prompt diagnosis and treatment, about 10% of patients with meningitis are prone to die and about 20% of those who survive may suffer from permanent disabilities [6–9]. In Africa, meningococcal, *H*. *influenzae* and pneumococcal meningitis have been reported as the most common laboratory confirmed cases in adults [3,4,8,9]. In sub-Saharan Africa, the region which is most affected by HIV worldwide, co-occurrence with meningitis is common [1,5,10]. The HIV infection has contributed to modifications in the aetiological profile of adulthood meningitis in African countries, including Mozambique [1,10,11], where *Cryptococcus neoformans* and *Mycobacterium tuberculosis* have become common causes of meningitis in adults living with HIV [11–19]. However, despite the improvement in the access to combined antiretroviral therapy (ART), mortality due to meningitis remains high [11–19]. The sub-optimal ART coverage and retention on ART in the African continent [20] are major causes of the high burden of meningitis in adults.

The worldwide mortality rate due to cryptococcal meningitis has been estimated at 181.100, of which, 75% (135 900/181.100) occur in sub-Saharan Africa [19]. Tuberculous meningitis (TBM) is considered to be the second leading cause of death by meningitis among adults in sub-Saharan Africa and more than 50% of these deaths occur in HIV-infected patients [19].

Bacterial meningitis can be prevented with the use of vaccines [21–24] and severe cryptococcal or TB meningitis can be avoided by a combination of either cryptococcal or TB screening and pre-emptive antifungal or TB therapy among HIV-infected adults [12]. In Mozambique, Hib and 10-valent pneumococcal conjugate vaccines (PCV) were introduced into the Expanded Program on Immunization (EPI) in 2009 and 2013, respectively [12]. Data from the EPI program, shows coverage for PCV10 and Hib vaccines of 97% and 90%, respectively.

Prompt diagnosis and effective laboratory confirmation of the aetiologic agent of meningitis is critical for initiation of proper treatment. However, this is often challenging, especially in limited-resource settings [16,25]. As such, Mozambique is expanding molecular and rapid diagnostic testing for early laboratory detection of the aetiologic agents of meningitis in the interest of a rapid initiation of appropriate treatment plans.

According to national protocols, on patients with suspected bacterial meningitis, broad-spectrum antibiotics are used to cover its main etiology, including intravenous (IV) cefotaxime 2g, every six hours or ceftriaxone 2g twice a day for 5 to 14 days, depending on their availability. In case of cephalosporin-resistant strains, treatment regimens consist of the use of the aforementioned cephalosporins combined with vancomycin 15–20 mg/Kg IV and 600 mg of rifampicin IV/orally 12 hourly, both for 7 to 14 days, twice a day. Current protocols for treatment of cryptococcal meningitis among patients living with HIV include the use of Amphotericin B and oral Flucytosine for seven days, followed by another week of administration of either orally or intravenous infusion Fluconazole [11,12].

Understanding the burden of meningitis in adults is critical for the definition of public health interventions in the interest of prevention, early diagnostic strategies, and proper treatment of meningitis [21]. This study was conducted with the aim of determining the burden of laboratory-confirmed fungal and bacterial meningitis among adults in 3 quaternary hospitals in Mozambique.

## Methods

### Study design, study setting and data collection

A retrospective cross-sectional study was conducted using data from the routine sentinel surveillance system for meningitis in Mozambique from January 2016 to December 2017. Sentinel surveillance system for meningitis in Mozambique has been established in three sentinel sites which are regional hospitals, namely Maputo Central Hospital (HCM) in Maputo Province, Beira Central Hospital (HCB) in Sofala Province and Nampula Central Hospital (HCN) in Nampula Province, located in the southern, central, and northern regions of the country, respectively as previously described by Nhantumbo *et al*., (2015) [26]. Also, according to this author, these three health facilities are quaternary reference hospitals for their region and offer several specialised services for all age groups [26].

[15]. According to data from the General Census of Population and Housing in Mozambique, the population of Maputo, Sofala and Nampula provinces are 2.507.098, 2.221.803 and 6.102.867 million, respectively [27] with an HIV prevalence of approximately 8.2%, 7.0% and 4.1% in 2015, respectively [28]. In Mozambique, healthcare is free of charge in the public sector and 70,9% of the national population is estimated to have access to a public healthcare.

All hospitalized adults (≥18 years old) at each of these three sentinel sites who met World Health Organization (WHO) case definition for Meningitis were consecutively enrolled in this study.

### Ethics approval and consent to participate

The study was approved by the Mozambican National Bioethics Committee (Ref #: 180/CNBS/20/IRB00002657). As per requirements of our routine sentinel surveillance for meningitis in Mozambique, written informed consent was applied to all participants over 18 years [26].

### Case definition

In Mozambique, case definition meningitis follows WHO guidelines [18]. Upon admission, patients with suspected meningitis are submitted to lumbar puncture and the standardized case report form (CRF) is completed [26].

Confirmed cases of meningitis is defined as the presence of bacterial or fungal pathogen identified either by culture, antigen detection, immunochromatography test or multiplex qPCR in the cerebrospinal fluid (CSF) [26,29].

### Sample and data collection

Basic demographic data such as age, gender, occupation and others were recorded using a standard case investigation form previously described by Nhantumbo *et al*., (2015) [26]. Nonetheless, an evaluation of the overall clinical presentation, as well as the HIV serological status and CD4 cell count was also taken into considerationSamples were collected at each sentinel site and were then sent to their respective microbiology laboratory for microbiological culture and antimicrobial susceptibility test (AST) as previously described by Nhantumbo *et al*., (2015) [26].

### Laboratory testing

#### Acute bacterial meningitis and Cryptococcal meningitis diagnostic

All CSF samples were first processed at the clinical laboratories from each sentinel site using Gram stain, India-ink test or CSF CrAg lateral flow assay (LFA) and culture as routine diagnostic tests [16,26]. Confirmatory testing was performed at the Reference Bacteriology and High-Risk Pathogens Laboratory at the National Institute of Health (NIH) in Mozambique, using culture and PCR methods. Cryptococcal meningitis was confirmed at the reference laboratory by culture and CrAg lateral flow assay andall positive samples for *S*. *pneumoniae*, *H*. *influenzae and N*. *meningitidis* were confirmed by qPCR while *Escherichia coli* and *Staphylococcus aureus* were confirmed by culture and latex agglutination antigen test.

#### Human immunodeficiency viruses testing

All patients were tested for HIV using the national algorithm for HIV testing. As per this algorithm, Determine HIV1/2 was used for initial screening of antibodies against HIV and Uni-gold HIV was used for confirmation of positive test result. The immunophenotyping of T lymphocytes was performed by flow cytometry using the FACSCalibur flow cytometer (Becton Dickinson, USA) and absolute values of total TCD4 lymphocytes were determined by the TRUcount method (Becton Dickinson, EUA).

#### Molecular detection of S. pneumoniae, H. influenzae and N. meningitides

Triplex qPCR assay was performed using a set of specific primers and probes targeting the three pathogens in a single multiplex reaction and FAM, Cy5, and HEX fluorophores for Cu-Zn superoxide dismutase gene *(sodC*) for *N*. *meningitidis*, autolysin gene (*lytA*) for *S*. *pneumoniae* and the protein D encoding gene (*hpd*) for *H*. *influenzae*, respectively [30]. Standard strains used as positive controls for specie identification were: *S*. *pneumoniae* ATCC 49619, *H*. *influenzae* ATCC 49247 and *N*. *meningitidis* ATCC13077. A no template negative controls (NTCs) was used in each reaction. All samples with cycle threshold (ct) value ≤ 35 were considered positive [30].

### Statistical analysis

Data entry was performed using Epi Info version 3.5.4 (CDC U.S.A.) and all analyses were conducted using R statistical software version 4.1.1 (Vienna, Austria). Results were reported as proportions and statistical significance differences were assessed using Fisher’s exact test and Pearson’s Chi-square. All variables with *p* value less than 0.05 were considered statistically significant.

## Results

### General characteristics of study participants

Between January 2016 and December 2017, a total of 1501 CSF samples were collected among adults clinically suspected of meningitis at three representative sentinel sites in the country. Of these CSF samples, 72.8% (1093/1501) were collected in 2016, while 27.2% (408/1501) in 2017. The median age of study participants was 36 years old (IQR 30–48 years) and 49.0% (736/1501) of them were male. More than half of the participants were from Maputo Central Hospital (69.2%, 1039/1501), while adults from Nampula Central Hospital and Beira Central Hospital comprised 12.7% (198/1501) and 18.1% (72/1501), respectively.

### Etiology of acute bacterial and fungal meningitis in adults in Mozambique between January 2016 and December 2017

**Fig 1:**
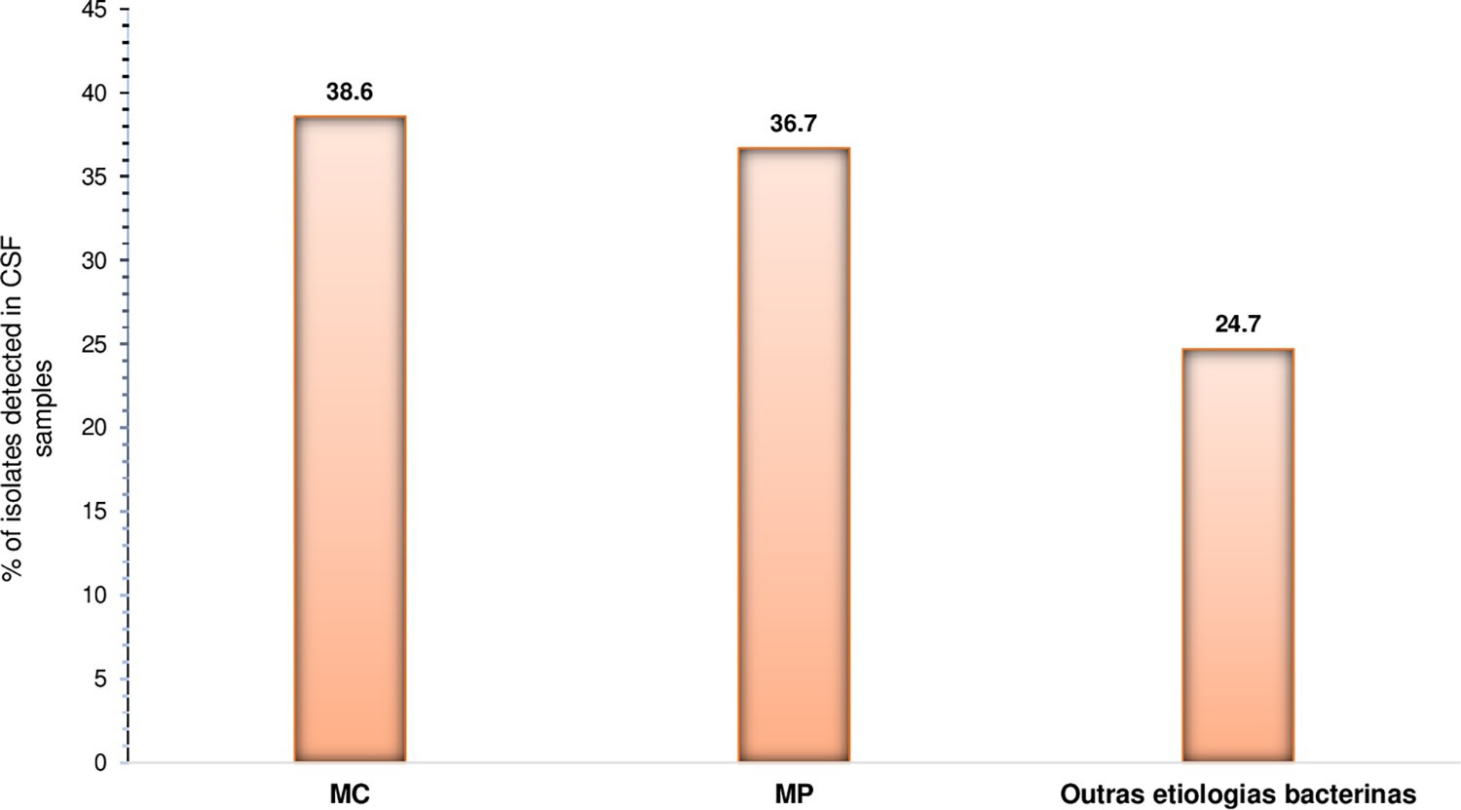
Proportion of confirmed bacterial and fungal meningitis cases in Mozambique in the period between January 2016 and December 2017. This figure shows the proportion of pathogens causing bacterial and fungal meningitis among adults in Mozambique in the period between January 2016 and December 2017.

During the two-year study period, 10.5% (158/1501) of the collected samples, were positive for bacterial or fungal meningitis, of which the majority, 38.6% (61/158), were identified as *Cryptococcus neoformans* followed by 36.7% (58/158) that were identified as *Streptococcus pneumoniae*. Other bacterial meningitis comprised 24.7% (39/158) of the total confirmed cases (see Fig 1), which include *Neisseria meningitidis* (8.9%; 14/158), *Escherichia coli* (6.3%; 10/158), *Haemophilus influenzae* (5.1%; 8/158) and *S*. *aureus* (4.4%; 7/158) (see Table 1). A total of 55 out of 61 patients with cryptococcal meningitis were HIV seropositive.

**Table 1:** Frequency of the pathogens causing bacterial and fungal meningitis among adults in Mozambique 2016–2017.

Type of isolate	Year		Total n (%)
	2016 n (%)	2017 n (%)	
*Cryptococcus neoformans*	40 (33.9)	21 (52.5)	61 (38.6)
*Streptococcus pneumoniae*	56 (47.5)	2 (5.0)	58 (36.7)
*Neisseria meningitidis*	9 (7.6)	5 (12.5)	14 (8.9)
*Escherichia coli*	4 (3.4)	6 (15.0)	10 (6.3)
*Haemophilus influenzae*	7 (5.9)	1 (2.5)	8 (5.1)
*Staphylococcus aureus*	2 (1.7)	5 (12.5)	7 (4.4)
**Total**	**118**	**40**	**158**

No statistically significant difference was observed in demographic characteristics (age group, gender and geographical location) regarding the aforementioned most frequent causes of meningitis (see Table 2).

**Table 2:** Demographic characteristics of patients with laboratory-confirmed fungal and bacterial meningitis in three sentinel sites, Mozambique, 2016–2017 (n = 158).

Variable	C. meningitis positive (n = 61)	C. meningitis negative (n = 1440)	Odds Ratio [95%CI]	*p*-value	P. meningitis positive (n = 58)	P. meningitis negative (n = 1443)	Odds Ratio [95%CI]	*p*-value	Other BM positive (n = 39)	Other BM negative (n = 1462)	Odds Ratio [95%CI]	*p*-value
**Age group**												
[18,25)	6 (9.8%)	174 (12.1%)	ref.	0.29	5 (8.6%)	175 (12.1%)	ref.	0.58*	3 (7.69%)	177 (12.11%)	ref.	0.39*
[25,35)	23 (37.7%)	545 (37.9%)	0.82 [0.30–1.92]		20 (34.5%)	548 (38.0%)	1.28 [0.51–3.88]		14 (35.90%)	554 (37.89%)	1.49 [0.48–6.52]	
[35,55)	23 (37.7%)	606 (42.1%)	0.91 [0.33–2.13]		26 (44.8%)	603 (41.8%)	1.51 [0.62–4.51]		16 (41.03%)	613 (41.93%)	1.54 [0.51–6.67]	
[>55)	9 (14.8%)	115 (8.0%)	0.44 [0.14–1.25]		7 (12.1%)	117 (8.1%)	2.09 [0.65–7.22]		6 (15.38%)	118 (8.07%)	3.00 [0.78–14.43]	
**Gender**												
Female	23 (40.4%)	631 (47.3%)	ref.	0.30	34 (59.7%)	620 (46.5%)	ref.	0.051	15 (39.47%)	639 (47.2%)		0.35
Male	34 (59.7%)	703 (52.7%)	0.75 [0.43–1.29]		23 (40.4%)	714 (53.5%)	0.59 [0.34–1.00]		23 (60.53%)	714 (52.8%)	1.37 [0.72–2.71]	
Missing	4	106			1	109			1	109		
**Hospital**												
HCB	6 (9.84%)	266 (18.47%)	ref.	0.19	9 (15.5%)	263 (18.2%)	ref.	0.74	2 (5.13%)	270 (18.5%)	ref.	0.06*
HCM	48 (78.69%)	991 (68.82%)	0.47 [0.18–1.02		40 (69.0%)	999 (69.2%)	1.17 [0.59–2.60]		33 (84.62%)	1006 (68.8%)	4.43 [1.34–27.42]	
HCN	7 (11.48%)	183 (12.71%)	0.59 [0.19–1.80]		9 (15.5%)	181 (12.5%)	1.45 [0.56–3.79]		4 (10.26%)	186 (12.7%)	2.90 [0.56–21.10]	
**Year**												
2016	40 (65.6%)	1053 (73.1%)	ref.	0.19	56 (96.6%)	1093 (72.8%)	ref.		22 (56.41%)	1071 (73.3%)		**0.02**
2017	21 (34.4%)	387 (26.9%)	0.70 [0.41–1.22]		2 (3.5%)	408 (27.2%)	0.09 [0.01–0.29]	**<0.001**	17 (43.59%)	391 (26.7%)	2.12 [1.10–4.01]	

Note: **BM**: Bacterial meningitis

*Fisher’s Exact Test.

**Fig 2:**
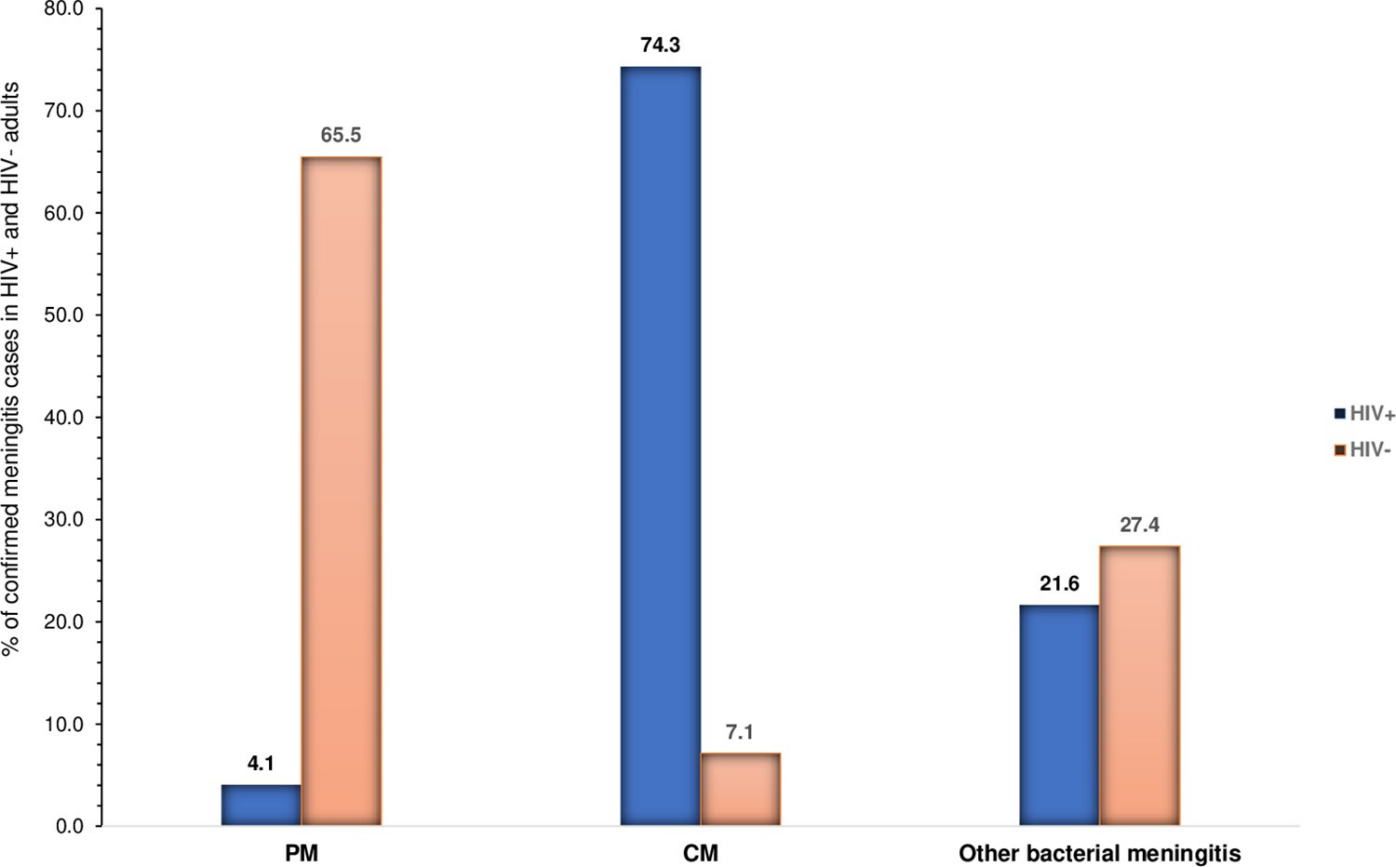
Proportion of Cryptococcal and pneumococcal meningitis in among HIV- infected and HIV- uninfected adults. Each bar represents the relative frequency of the main cause of meningitis among two groups of patients. **PM**: Pneumococcal meningitis; **CM**: Cryptococcal meningitis.

Frequency of cryptococcal meningitis was significantly higher among HIV- infected adults as opposed to HIV-uninfected adults (74.3%, 55/74 versus 7.1%, 6/84; *p* < 0.001). Nevertheless, frequency of pneumococcal meningitis was higher among HIV- uninfected adults as opposed to HIV-infected adults (65.5%, 55/84 versus 4.1%, 3/74; *p* < 0.001) (see Fig 2).

### Predicting variables associated with meningitis infection

Table 3 shows the univariate and multivariate logistic regression analysis results. Multivariate logistic regression analysis found that meningitis infection was significantly associated with CD4 count < 100 cells/μL (adjusted Odds Ration (aOR) with 95% CI: 59.0, 18.80–265.27, *p* = 0.001) (see Table 3).

**Table 3:** Univariate and multivariate logistic model for predictors of meningitis among adult patients in Mozambique, 2016–2017.

Variable	Confirmed meningitis (n = 158)			Unadjusted OR [95% CI]	*p*-value	Adjusted OR [95% CI]	*p*-value
** **	**n/N**	**%**	**95% CI**	-	-	-	-
**Age (years)**							
**Median (IQR)**		36.0	[30.0–47.5]	-	-	-	-
**Age (group)**							
**18–25**	14	8.9	[4.7–15.9]	ref	-	-	-
**25–35**	50	31.6	[25.2–42.8]	1.3 [0.74–2.52]	0.37	-	-
**35–55**	68	43.0	[31.4–49.7]	1.4 [0.77–2.59]	0.31	-	-
**>55**	26	16.5	[10.6–24.8]	2.6 [1.26–5.33]	**0.01**	1.3 [0.16–12.04]	0.79
**Sex**							
**Male**	80	52.6	[44.4–60.8]	1.0 [0.70–1.39]	0.94	-	-
**Female**	72	47.4	[39.2–55.6]	ref	-	-	-
**Missing**	6	-	-		-	-	-
**CD4 count**							
**<100 cells/μL**	52	70.3	[58.4–80.1]	58.0 [18.80–265.27]	**0.001**	59.0 [18.80–265.27]	**0.001** ^*^
**≥100 cells/μL**	22	29.7	[19.9–41.6]	ref	-	-	-
**Year**							
**2016**	118	74.7	[67.2–81.3]	ref	-	-	-
**2017**	40	25.3	[18.7–32.8]	0.89 [0.60–1.3]	0.58	-	-
**Sentinel Site**							
**HCB**	17	10.8	6.4–16.7	ref	-	-	-
**HCM**	121	76.6	69.2–82.9	1.97 [1.20–3.46]	**0.01**	0.29 [0.06–1.45]	0.12
**HCN**	20	12.7	7.9–18.9	1.76 [0.89–3.50]	0.099	-	-

Note: **CI**: Confidence interval; **CSF**: Cerebrospinal fluid; **OR**: Odds ratio.

*Fisher’s Exact Test.

There was no significant association with meningitis infection regarding the age group, as well as between meningitis infection and geographical location. Although, the burden of disease in HCM was slightly high (see Table 3).

## Discussion

In this study we report, for the first time, the burden of bacterial and fungal meningitis among adults in three most populated provinces of Mozambique. There have been a number of previous studies of bacterial and fungal meningitis in adults in the country, but they were all conducted in a small village in southern part of the country [31,32].

Results of this study show that *Cryptococcus neoformans* is responsible for 38.6% of all laboratory-confirmed cases, thus making it the leading cause of meningitis among adults. These are similar to previously reported findings from other Sub-Saharan African countries, having found frequencies ranging from 60.5% to 71.6% [1,11,16,33–38].

The second most common cause of meningitis found through our study was *Streptococcus pneumoniae*, which is responsible for 36.7% of the infections in the country. This is also in accordance with results from previous studies conducted in adults in other African studies [39–42]. Because Mozambique introduced the pneumococcal conjugate vaccines (PCVs) into the Extended Program on Immunization (EPI) for pneumococcal disease in 2013, it has been deemed important to understand its impact on meningitis in unvaccinated adults. Although, data from other countries in sub-Saharan Africa show a decline on the impact of this disease on children [24,39,43,44], as well as, is on unvaccinated adults including those who are HIV-positive [40,45,46] this is yet to be investigated in Mozambique.

When stratified by HIV serostatus, cryptococcal meningitis was more common among HIV- infected adults when compared to HIV-uninfected adults, as corroborated by similar findings reported in other African countries with high HIV prevalence [1,36,47–51]. In sub-Saharan African, several factors may contribute to the high burden of cryptococcal meningitis, which include late HIV diagnosis and ART initiation, poor ART adherence, poor retention in care and late CrAg screening [12,51].

Findings from this study are a strong reminder of the urgent need for implementation of low-cost, point-of-care lateral flow assays (LFAs) CrAg, which are not yet a part of the routine screening among HIV infected patients in Mozambique. In 2014, a pilot implementation was conducted in three out of eleven provinces, namely, Gaza, Sofala and Cabo Delgado, but unfortunately it did not go any further. Current evidence shows that in areas with high HIV prevalence, CrAg screening in HIV infected patients with a CD4 Count ≤ 100 cells/μL is of paramount importance to provide rapid initiation of treatment leading to better patient outcomes [12,45]. World Health Organization (WHO) and the US Centre for Disease Prevention and Control (US-CDC) also recommend screening of *Cryptococcus neoformans* among HIV infected patients with low CD4 T cell count [21,52].

Despite the high burden of cryptococcal meningitis in sub-Saharan Africa, the access to life saving ART and antifungal medicines to treat cryptococcal meningitis remains limited [49,51,53,54]. For instance, in Mozambique, the coverage of ART in people living with HIV is still below 70% [55], highlighting that a large number of HIV infected patients are still at high risk of poor outcome due to the risk of developing cryptococcal meningitis.

We also investigated predictors for meningitis in adults in Mozambique and found that CD4 cell count < 100 cells/μL had strong association (aOR = 59, *p* = 0.001) with meningitis, as also reported in other countries [1,11,13,49–51,54–61].

We would like to acknowledge some limitations of our study, which may influence the interpretation of the results. For instance, our data was collected at three of the largest hospitals in Mozambique, which may raise some concerns regarding representativeness. Secondly, the poor quality of information recorded in the case investigation forms limited our ability to assess the outcomes among HIV- infected adults and HIV-uninfected adults. Finally, *Mycobacterium tuberculosis* testing was not performed because the approved protocol for the meningitis surveillance system in Mozambique does not include tuberculous meningitis, but our protocol is under revision in order to include these pathogens.

## Conclusion

Our study provides, for the first time, evidence on the burden of cryptococcal and bacterial meningitis among adults in three provinces in Mozambique, which found that cryptococcal meningitis is the leading cause of meningitis among adults. Moreover, patients with CD4 count < 100 cells/μL are at highest risk of suffering of meningitis, suggesting that there is an urgent need of rapid rollout of Cryptococcal Antigen (CrAg) Lateral Flow Assay in Mozambique for early detection of *Cryptococcus neoformans*, in order to improve the outcome of HIV infected patients in Mozambique.

## Abbreviations

ART: antiretroviral therapy
ATCC: american type culture collection
CDC: Centers for Disease Control and Prevention
CrAg: cryptococcal antigen
CRF: case report form
CSF: cerebrospinal fluid
ct: cycle threshold
HCB: Beira central hospital
HCM: Maputo central hospital
HCN: nampula central hospital
HIV: human immunodeficiency virus
INS: National Institute of Health
IBD-PV: invasive bacterial diseases preventable vaccines
IQR: interquartile range
LFA: lateral flow assay
LP: lumbar puncture
qPCR: multiplex qualitative polymerase chain reaction
PCV-10: ten-valent conjugate vaccine
PCV-13: thirteen -valent conjugate vaccine
TBM: Tuberculous meningitis
UFCSPA: Universidade Federal de Ciências de Saúde de Porto Alegre
WHO: World Health Organization
